# Long-Term Experience of Chinese Calligraphic Handwriting Is Associated with Better Executive Functions and Stronger Resting-State Functional Connectivity in Related Brain Regions

**DOI:** 10.1371/journal.pone.0170660

**Published:** 2017-01-27

**Authors:** Wen Chen, Yong He, Yang Gao, Cuiping Zhang, Chuansheng Chen, Suyu Bi, Pin Yang, Yiwen Wang, Wenjing Wang

**Affiliations:** 1 State Key Laboratory of Cognitive Neuroscience and Learning, Beijing Normal University, Beijing, China; 2 Department of Psychology and Social Behavior, University of California, Irvine, California, United States of America; 3 IDG/McGovern Institute for Brain Research, Beijing Normal University, Beijing, China; 4 School of International Journalism and Communication, Beijing Foreign Studies University, Beijing, China; 5 School of Arts and Media, Beijing Normal University, Beijing, China; 6 Conservation Department, National Palace Museum, Beijing, China; Peking University, CHINA

## Abstract

Chinese calligraphic handwriting (CCH) is a traditional art form that requires high levels of concentration and motor control. Previous research has linked short-term training in CCH to improvements in attention and memory. Little is known about the potential impacts of long-term CCH practice on a broader array of executive functions and their potential neural substrates. In this cross-sectional study, we recruited 36 practitioners with at least 5 years of CCH experience and 50 control subjects with no more than one month of CCH practice and investigated their differences in the three components of executive functions (i.e., shifting, updating, and inhibition). Valid resting-state fMRI data were collected from 31 CCH and 40 control participants. Compared with the controls, CCH individuals showed better updating (as measured by the Corsi Block Test) and inhibition (as measured by the Stroop Word-Color Test), but the two groups did not differ in shifting (as measured by a cue-target task). The CCH group showed stronger resting-state functional connectivity (RSFC) than the control group in brain areas involved in updating and inhibition. These results suggested that long-term CCH training may be associated with improvements in specific aspects of executive functions and strengthened neural networks in related brain regions.

## 1. Introduction

Chinese calligraphy has a long history, originated from oracle-bone writing (*chia ku wen*) and evolved into subsequent five main forms, including seal script (*chuan shu*), clerical script (*li shu*), running script (*hsing shu*), grass writing (*tsao shu*), and model script (*kai shu*) [1]. To master any style of Chinese calligraphy is a difficult task and requires years of practice, which includes learning the precise creation of each stroke, the composition of the whole piece, and the rhythm of writing and associated breathing [2].

The particular demands of Chinese calligraphic handwriting (CCH) on mental resources have intrigued psychologists since the 1970s [3]. Researchers have found that the act of brush-writing is associated with calligraphers’ physiological changes [4, 5] and brain activity [6, 7]. Physiological changes include decelerated respiration, slower heart rate, decreased blood pressure, and reduced muscular tension [4, 5]. These changes are similar to those resulting from relaxation training [8] or mindful meditation [9, 10], suggesting that CCH promotes relaxation and attention/concentration, which are further related to executive functions (EFs) [11, 12]. In terms of neural correlates of CCH, an EEG study [13] found that CCH training increased theta wave, which has been associated with working memory (a key component of EF) in previous studies [14–16]. Finally, indirect evidence linking CCH to EFs also came from the beneficial effect of short-term CCH training in children with attention deficiency (attention-deficit/hyperactivity disorder) [17]. These children are known to have deficits in EFs [18–21], but they were helped by CCH training, perhaps via improved EFs.

In sum, previous research has provided some evidence linking CCH to EFs. No study to our knowledge, however, has systematically examined the effects of long-term CCH on various components of EFs and related brain connectivity.

Based on Miyake’s conceptualization [22], EFs have three components: updating (or WM), shifting (or cognitive flexibility), and inhibition (or control). Previous studies have identified the brain networks related to these three components of EFs. Specifically, previous fMRI studies have showed that both dorsolateral frontal cortex (dlPFC) [23–25] and superior frontal cortex [26, 27] play a key role in WM. One training study found that the activity of the prefrontal and parietal cortices was increased after 5 weeks of WM training [28]. Another study found that task-related effective connectivity in the fronto-parietal networks was enhanced by an intensive training using the N-back task [29]. Most relevant to this study, Hampson et al. (2006) further found that WM performance was related to RSFC between the PCC and MFG/ACC [30]. Inhibition is shown to involve a common neural network that includes the prefrontal cortex and the anterior cingulate cortex [31–33]. The right inferior prefrontal gyrus (IFG) is particularly critical for behavioral inhibition [34, 35]. In terms of cognitive flexibility, the fronto-striatal brain network has been found to be involved in task switching or shifting [36, 37], and the left inferior frontal junction (IFJ) also plays a hub role in task-switching [38].

The present study included 36 individuals with long-term CCH experience (at least five years) and 50 controls (less than five months of CCH experience). Both behavioral data (with three sub-tests to measure the three components of EFs) and resting-state brain data were collected. We hypothesized that the group with long-term CCH experience would show better EF and stronger RSFC in EF-related brain areas.

## 2. Materials and Methods

### 2.1 Participants

Participants were 36 students from the calligraphy major and 50 controls from other social sciences and humanities majors at the Beijing Normal University, Beijing, China. The CCH participants had had at least five years of formal training in CCH and the controls had no special CCH training and no more than a few months of basic school experience with CCH. All participants were right-handed native Chinese speakers. A written consent form was obtained from each participant after a full explanation of the study procedure. This study was approved by the Institutional Review Board of the State Key Laboratory of Cognitive Neuroscience and Learning at Beijing Normal University, China. Subjects were compensated for their time.

### 2.2 Neuropsychological measures

In the present study, we collected demographic information and administered an IQ test and three neuropsychological measures of the three components of EF. We measured updating with the Corsi block test, shifting with a cue-target paradigm task, and inhibition with the Stroop color-word test.

#### 2.2.1 Raven’s Advanced Progressive Matrices test

All participants were asked to complete both Set I and Set II of the *Raven’s Advanced Progressive Matrices* (APM) test [39]. The standard instructions were read aloud by the experimenter, and the time limits were 5 minutes and 40 minutes for Set I and Set II, respectively. The scores from Set II were used to index IQ.

#### 2.2.2 Cue-target paradigm task

Shifting was measured by a cue-target task—the covert attentional orienting task [40]. We used a computerized version that included 10 practice trials and 120 formal trials. In this task, a cross was placed at the center of the computer screen, and two boxes were placed on the left and right of the cross. A cue randomly appeared in the left or right box. A stimulus onset asynchrony (SOA) of 50 ms, 250 ms, or 950 ms separated the cue and the target. Participants responded to the peripheral target while remaining visually fixated at the center of the screen. The targets were preceded by a visual cue, which might occur in the same location as the subsequent target (valid trials) or in a location contralateral to the target (invalid trials). In the current study, we were not interested in the ‘‘inhibition of return” attentional mechanism that was prevalent at longer SOA periods (>300ms) [41, 42], and therefore, only the responses to SOAs of the 50 ms (SOA_50) and 250 ms (SOA_250) trials were analyzed. Shifting (or attentional flexibility) was indexed by the validity effect [43, 44], which was calculated by subtracting the valid cue reaction times from the invalid-cue reaction times.

#### 2.2.3 Corsi block test

The Corsi block test [45] was used to measure spatial WM. Participants were asked to remember varying sequences of spatial locations and to recall them in forward and backward order. The forward recall test measures visuo-spatial short-term memory, and the backward recall measures visuo-spatial WM [46–48], so only backward recall scores (the sequence length times the total number of correct trials out of the total 14 trials) were used in this study.

#### 2.2.4 Stroop color-word test

The Stroop color-word test is one of the most often used experimental paradigms to measure inhibitory control. Based on the standard Stroop color-word test [49], we created a computerized version that included three experimental blocks (reading the color word, naming the color, and naming the color of a word printed in an incongruent color) with 12 practice trials and 84 formal trials each. We recorded the mean reaction time (RT) and accuracy rate (ACC) of the three experimental blocks and calculated the inverse efficiency score (IE = -RT/accuracy). Finally, the interference score for time (IS time) (calculated by subtracting the average time needed to complete the word-naming and color-naming trials’ RT from the incongruent trials’ RT [50]) and IE difference score (calculated by subtracting the color-naming trials’ IE from the incongruent trials’ IE) were used as the Stroop interference scores.

### 2.3 Brain imaging data collection and preprocessing

#### 2.3.1 fMRI data acquisition

MRI data were collected using a SIEMENS TRIO 3-Tesla scanner in the Brain Imaging Center of Beijing Normal University. Participants lay supine with their heads snugly fixed by a belt and foam pads to minimize head motion. Each participant underwent an eight-minute resting-state fMRI (RS-fMRI) scanning session and a 3D anatomic session. During the RS-fMRI session, the participants were instructed to keep their eyes closed, be as still as possible, and not to think about anything in particular. Images were obtained with the following parameters: 33 axial slices, thickness/gap = 3.5/0.7 mm, matrix size = 64×64, repetition time (TR) = 2000 ms, echo time (TE) = 30 ms, flip angle = 90°, field of view (FOV) = 200×200 mm^2^. The 3D T1-weighted magnetization- prepared rapid gradient echo (MPRAGE) image was acquired with the following parameters: 144 sagital slices, slice thickness/gap = 1.3/0.65 mm, TR = 2530 ms, TE = 3.39 ms, inversion time (Ti) = 1100 ms, flip angle = 7°, FOV = 256×256 mm^2^, matrix size = 256×192.

#### 2.3.2 Image preprocessing

Data Processing Assistant for Resting-State fMRI (DPARSF, http://rfmri.org/DPARSF) [51] was used to preprocess the RS-fMRI data. Steps included: (1) discarding the first 10 volumes; (2) correcting for within-scan acquisition time differences between slices and head motions; (3) coregistering the T1 image to the mean functional image using a linear transformation; (4) segmenting the coregistered T1 images into gray matter, white matter, and cerebrospinal fluid; (5) normalizing the head motion corrected functional images to the standard template using the transformation matrix estimated from T1 segmentation and reslicing them to 3 mm isotropic resolution; (6) smoothing the images with FWHM = 4 mm; (7) linear detrending to reduce the effects of low-frequency drift; and (8) regressing nuisance signals including the Friston 24 head motion parameters, cerebrospinal fluid signal, and white matter signal from the data. Finally, temporal band-pass filtering (0.01~0.1 Hz) was used to reduce high-frequency physiological noise.

### 2.4 Data analysis

We performed two-sample *t* tests for the three behavioral tasks. For the brain data, one participant of the CCH group and four participants of the control group were excluded because of excessive head motion (>2 mm), yielding a final sample of 31 CCH and 40 control participants with valid brain imaging data. We used the REST V1.8 (http://restfmri.net/forum/REST_V1.8) [52] for the FC analysis. Mean time series for regions of interest (ROIs) were extracted, and voxel-wise correlation analysis was then used to generate the FC map. Correlations coefficients were converted into z map by Fisher’s r-to-z transformation to improve the normality.

Specifically, nine brain regions (r = 6.00mm) were selected as seed regions of interest (seed ROIs) in this study (Table 1). Four of these ROIs have been linked to WM, including bilateral dorsolateral frontal cortex (dlPFC, Brodmann area BA9) [25, 27] and superior frontal gyrus (SFG, BA 10) [26]. Two ROIs, left/right inferior frontal gyrus (IFG, BA 44)[53], have been linked to inhibition control. One ROI in the left inferior frontal junction (IFJ, BA 6)[38] has been linked to shifting. Finally, two ROIs in the anterior cingulate cortex (ACC, BA 32) have been linked to all three components of EF [54, 55].

**Table 1:** MNI coordinates of seed ROIs.

ROI	Dimension of Efs	Side	Seed location	BA	MNI		
1	WM	L	dlPFC	9/46	-38	34	43
2		R	dlPFC	9/46	42	34	42
3		L	SFG	10	-7	58	-11
4		R	SFG	10	8	58	-12
5	Inhibition	L	IFG	44	-48	10	24
6		R	IFG	44	51	11	23
7	Shifting	L	IFJ	6	-41	3	31
8	Related to all three components of EF	L	ACC	32	-6	33	23
9		R	ACC	32	9	44	23

Note: L: left hemisphere; R: right hemisphere; dlPFC: dorsolateral frontal cortex; SFG: superior frontal gyrus; IFG: inferior frontal gyrus; IFJ: inferior frontal junction; ACC: anterior cingulate cortex.

FCs between the nine ROIs and the whole brain were calculated and Z value maps were generated for group analysis in SPM8, with age, gender, and IQ as covariates and a topological FDR (p<0.01) correction. Brain areas showing strong RSFC with the seed regions were identified as target ROIs. The averaged RSFC between the target ROIs and the seed regions were then correlated with performance on the EF tasks to confirm these connections’ roles in EF.

## 4. Results

### 4.1 Behavioral results

The CCH and control groups did not differ in terms of gender (χ^2^ = 0.251, *p* = 0.671), age (*t* = -0.132, *p* = 0.895), years of education (*t* = -0.147, *p* = 0.884), and IQ (*t* = -0.658, *p* = 0.513). The CCH practitioners had 5–20 years of experience (M = 10.69 years, SD = 3.55), started practicing at 5–20 years of age (M = 9.14 years, SD = 4.05), and practiced CCH on average for 0.50–7.00 hours per day (M = 2.44 hours, SD = 1.38) (Table 2). In terms of the type of scripts, most participants had experience with at least two or three types of scripts, with the regular and the running scripts being the most common.

**Table 2:** Demographic and other information about the CCH group and control group.

Variables	CCH	Controls	*t or χ2*	*p*
N (Male/Female)	36 (16/20)	50(17/33)	0.251	0.617
Age (mean±SD in year)	21.31±2.16 (18.08~26.42)	21.28±2.40 (17.17~28.42)	-0.132	0.895
Handedness (% right handed)	100	100		
Education (mean ±SD in year)	14.44±2.08 (9~19)	14.03±1.84 (12~18)	0.147	0.884
APM	26.59±3.73 (18~33)	25.54±8.23 (20~35)	-0.658	0.513
Years of practicing CCH (mean±SD in year)	10.69±3.55 (5~20)			
The age of starting practicing CCH (mean±SD in year)	9.14±4.05 (5~20)			
Mean hours of practicing CCH per day (mean±SD in hours)	2.44±1.38 (0.50~7.00)			

Note: APM: Raven’s Advanced Progressive Matrices (APM) test. Range of scores are presented in parentheses.

For the cue-target task, we found neither excessively slow responses (>1500 ms) due to inattentiveness or fatigue nor excessively fast (<100 ms) responses due to anticipatory errors [56]. Non-valid trials (i.e., negative validity effect trials and outliers beyond three standard deviations) were deleted. The CCH and control groups did not differ significantly in attentional flexibility (shifting) in the 50 ms trials (*t* = -0.250, *p* = 0.803) or the 250 ms trials (*t* = 0.649, *p* = 0.518) (Table 3 and S1 Table). In terms of working memory or updating, CCH subjects performed significantly better than the controls (*t* = 2.276, *p* = 0.026) (Table 3 and S1 Table). Finally, the CCH showed better inhibition than the controls, as shown by the former’s shorter RT on the incongruent trials of the Stroop color-word test (see Table 3 and S1 Table), lower IS time, *t* = -8.912, *p* < 0.001, and higher IE difference score *t* = 7.410, *p* < 0.001.

**Table 3:** Group differences in the cue-target paradigm task, Corsi block test, and Stroop color-word test.

Tasks	CCH Group (N = 36)	Control Group(N = 50)	*t*	*p*
*Cue-target paradigm task*				
**VE_50(ms)**	28.66±16.38	30.15±32.00	-0.250	0.803
**VE_250(ms)**	28.44±24.09	21.42±61.00	0.649	0.518
*Corsi block test*				
**Forward score**	54.41±21.46	42.64±15.32	1.452	0.151
**Backward score**	48.09±17.51	35.39±13.19	2.276	0.026
*Stroop Color-Word test*				
**IS time**	100.52±62.02	251.30±79.17	-8.912	0.000
**IE difference score**	-72.12±68.12	-205.46±78.61	7.410	0.000

Note: VE_50 = validity effect of SOA_50, VE_250 = validity effect of SOA_250; IS time = the interference score for time; IE = inverse efficiency score. IE difference score was calculated by subtracting the color-naming trials’ IE from the incongruent trials’ IE. The p values were not corrected for multiple tests.

### 4.2 RS-fMRI results

**Fig 1:**
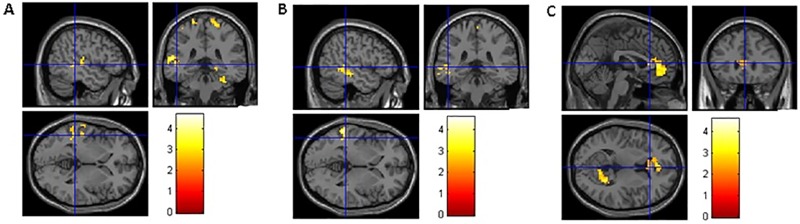
Brain areas showing stronger RSFC with seed ROIs related to WM. A and B show brain areas with stronger RSFC with left dlPFC and right dlPFC, and the coordinates of the cross in A and B are the same, [–48, –36, 0]. C shows brain areas with stronger RSFC with left dlPFC, and the coordinates of the cross in C are [0, 27, 9].

**Fig 2:**
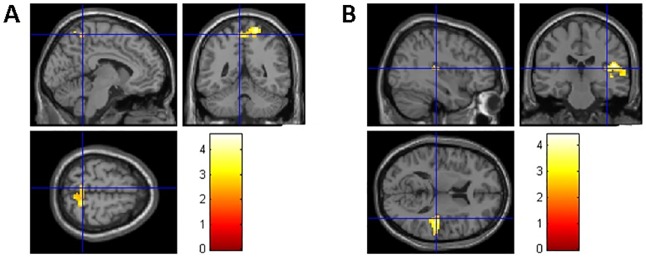
Brain areas showing stronger RSFC with left (A) and right SFG (B) seeds related to inhibition. The coordinates of the cross in A and B are [–6, –48, 63] and [39,–24, 12], respectively.

**Fig 3:**
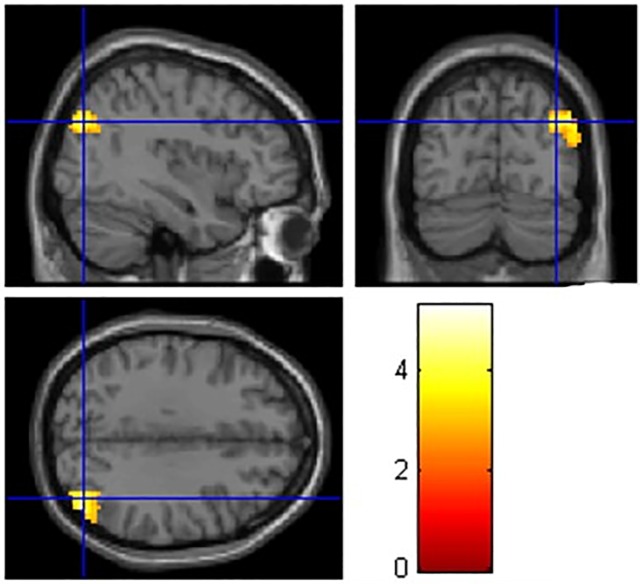
Brain areas showing stronger RSFC with left IFG related to shifting. The coordinates of the cross are [39,–75, 33].

**Fig 4:**
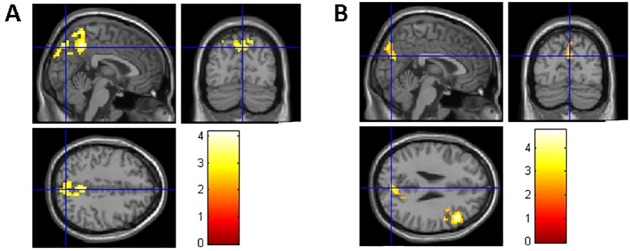
Brain areas showing stronger RSFC with left ACC (A) and right ACC (B) seeds related to all three components of EF. The coordinates of the cross in A and B are [3,–75, 42] and [3,–78, 27], respectively.

As hypothesized, we found that participants with long-term CCH experience showed stronger RSFC related to the brain areas involved in WM, inhibition control, and shifting (Table 4). Specifically, stronger RSFC for the CCH group than the control group were found (a) between the left dlPFC seed and the following areas: the fusiform gyrus (FFG), postcentral gyrus (PstCG), precentral gyrus (PCG) in the right hemisphere, and superior temporal gyrus (STG), middle temporal gyrus (MTG), PstCG, PCG and Heschl’s gyrus (HG) in the left hemisphere (Fig 1A); (b) between the right dlPFC seed and the following areas: MTG and inferior temporal gyrus (ITG) in the left hemisphere and bilateral precuneus (Fig 1B); (c) between the left SFG seed and anterior cingulate cortex (ACC) and angular gyrus (AG) in the left hemisphere and medial orbitofrontal cortex (MOFC), precuneus, and the primary visual cortex such as calcarine cortex(CC) in the right hemisphere (Fig 1C); (d) between the left IFG seed and right PstCG, superior parietal gyrus (SPG) and precuneus (Fig 2A); (e) between the right IFG seed and right STG (Fig 2B); (f) between the left IFJ seed and the parahippocampal gyrus (PHG), hippocampus, and ACC in the left hemisphere, and precuneus, MOG, MTG, PstCG, and SPG in the right hemisphere (Fig 3); and (g) between the bilateral ACC seeds (bilateral precuneus) and right IFG, MTG, PCG, superior occipital gyrus (SOG), thalamus, caudate, cuneus, and rolandic operculum (RO) (Fig 4). No group differences were found for RSFC with the right SFG seed.

**Table 4:** Brain areas showing stronger RSFC among CCH participants than controls based on ROI seeded FC analyses with topological FDR (p < 0.01) correction.

ROI seeds	Cluster size	Peak (MNI)			Side	Cluster location	Brodmann areas (BA)	Peak T
		X	Y	Z				
***WM***								
L_dlPFC	149	42	-15	-27	R	FFG	20	4.7
		42	-24	-24	R	FFG	20	3.51
		27	-42	-15	R	FFG	37	3.49
	251	18	-27	78	R	PstCG/PCG	4	3.97
		-21	-24	60	L	PCG	6	3.68
		-21	-36	75	L	PstCG	4	3.59
	116	-57	-39	12	L	MTG/STG	22/42	3.89
		-45	-21	12	L	HG	48	3.19
		-51	-24	3	L	STG	48	3.17
R_dlPFC	129	-60	-39	-3	L	MTG	21	4.62
		-66	-42	-15	L	ITG	20	3.13
	139	6	-63	63	R	Precuneus	7	3.58
		3	-48	66	R	Precuneus	5	3.58
		-9	-69	63	L	Precuneus	7	3.37
L_SFG	308	15	51	-3	R	MOFC	10	5.01
		6	36	-9	R	MOFC	11	4.51
		-3	39	6	L	ACC	32	3.99
	184	-42	-72	48	L	AG	7	4.41
		-45	-57	30	L	AG	39	3.03
	169	24	-54	9	R	CC	19	3.71
		24	-63	18	R	Precuneus	18	3.64
		15	-57	12		CC	17	3.55
***Inhibition***								
L_IFG	163	18	-48	69	R	SPG	5	4.63
		15	-39	72	R	PstCG	3	3.81
		9	-51	66	R	Precuneus	5	3.32
R_IFG	167	72	-33	6	R	STG	22	4.42
		48	-24	18	R	STG	48	4.27
		66	-27	6	R	STG	21	3.94
***Shifting***								
L_IFJ	123	-18	-12	-21	L	PHG/Hippocampus	35	5.3
		-27	-21	-24	L	PHG	30	4.02
		-18	-6	-27	L	PHG	28	3.92
	148	21	-45	72	R	SPG	1	4.49
		3	-51	60	R	Precuneus	5	3.4
		27	-39	69	R	PstCG	2	3.3
	170	45	-78	33	R	MOG	39	4.33
		60	-63	21	R	MTG		2.9
	280	12	30	-15	R	Rectus	11	3.81
		-12	33	-3	L	ACC	11	3.73
***Al three components of EFs***								
L_ACC	477	0	-51	45	L	Precuneus		4.18
		6	-78	48	R	Precuneus	7	3.85
R_ACC	283	48	21	27	R	IFG	48	4.77
		42	0	33	R	PCG	6	3.25
	155	15	-21	12	R	Thalamus		3.97
		9	9	6	R	Caudate		3.79
	128	48	-48	18	R	MTG	21/41	4.03
		57	-57	12	R	MTG	37	3.23
		51	-27	21	R	RO	48	3.01
	130	9	-72	27	R	Cuneus		3.65
		21	-63	36	R	SOG	7	3.37

Note: L: left hemisphere; R: right hemisphere; FFG: fusiform gyrus; PstCG: postcentral gyrus; PCG: precentral gyrus; MTG: middle temporal gyrus; STG: superior temporal gyrus; HG: Heschl’s gyrus; ITG: inferior temporal gyrus; MOFC: medial orbitofrontal cortex; ACC: anterior cingulate cortex; AG: angular gyrus; CC: corpus callosum; SPG: superior parietal gyrus; PHG: parahippocampal gyrus; MOG: middle occipital gyrus; MTG: middle temporal gyrus; IFG: inferior frontal gyrus; RO: rolandic operculum; SOG: superior occipital gyrus.

Finally, partial correlation analyses showed that WM had positive correlations with averaged RSFC between WM-related seeds and two target ROIs, FFG (*r* = 0.522, *p* = 0.007) and AG (*r* = 0.391, *p* = 0.054); and between seed ROIs related to all three components of EFs and two target regions, the thalamus/caudate (*r* = 0.398, *p* = 0.049) and the cuneus/SOG (*r* = 0.483, *p* = 0.015). The IS time had a negative correlation with averaged RSFCs between inhibition-related seeds and the target region of STG (*r* = -0.512, *p* = 0.009). The VE_250 had positive correlations with averaged RSFCs between shifting-related seed ROIs and the rectus/ACC (*r* = 0.434, *p* = 0.030); and between seed ROIs of all three components and two target regions, the thalamus/caudate (*r* = 0.406, *p* = 0.044) and the cuneus/SOG (*r* = 0.522, *p* = 0.007) (Table 5 and S2 Table).

**Table 5:** Partial correlations between behavioral measures and averaged RSFC between seed ROIs for a given component of EF and target brain areas of the CCH group after controlling for age, gender, and IQ.

Seed ROIs for components of EF	Behavioral measures	Side	Target brain areas	*r*	*p*
**WM** (L_dlPFC and L_SFG)	WM	R	FFG	0.522	0.007
	WM	L	AG	0.391	0.054
**Inhibition** (R_IFG)	IS	R	STG	-0.512	0.009
**Shifting** (L_IFJ)	VE_250	R/L	Rectus/ACC	0.434	0.03
**All three components of EFs** (R_ACC)	WM	R/R	Thalamus/caudate	0.398	0.049
	WM	R/R	Cuneus/SOG	0.483	0.015
	VE_250	R/R	Thalamus/caudate	0.406	0.044
	VE_250	R/R	Cuneus/SOG	0.522	0.007

Note: L: left hemisphere; R: right hemisphere; FFG: fusiform gyrus; AG: angular gyrus; STG: superior temporal gyrus; ACC: anterior cingulate cortex; SOG: superior occipital gyrus; VE_250 = validity effect of SOA_250; IS time = the interference score for time. The p values were not corrected for multiple tests.

## 5. Discussion

The current study explored the association between long-term experience with CCH and executive functions (EFs), including attentional flexibility, working memory, and inhibitory control. Results indicated that individuals with at least five years of CCH experience performed better than did the controls on two of the tests: one tapping working memory and the other inhibition. These results extended earlier work about short-term CCH training’s effects on cognitive abilities [17, 57, 58]. Moreover, we found that CCH participants showed stronger RSFC than did the control group across a number of brain regions, especially those related to EFs and the default mode network (DMN), visual processing network (VPN), primary somatomotor network (PSN), and basal ganglia.

There are several possible explanations/mechanisms of the association between CCH training and better EFs (especially inhibition and WM). First, CCH training has been shown to result in physiological relaxation and concentration, which benefit WM [59, 60] and inhibition [61]. Second, to master one or more styles of Chinese calligraphy is difficult and requires years of learning the precise creation of each stroke, the composition of the whole piece, and the rhythm of writing and associated breathing [2]. This type of training is similar to EF-training programs [12] in their reliance on (and thus providing challenges to) core EFs such as inhibition and WM. CCH training’s benefit for working memory is probably also due to the fact that Chinese calligraphic writing requires a continuous act of writing, rather than the stroke-by-stroke writing in daily life. Calligraphers have to keep in mind not only the characters but also their specific spatial layout and constantly monitor the remaining space so the end product is beautifully arranged [62, 63]. This writing process puts a high demand on WM resources [64], which is especially the case during early years of training. In sum, practicing CCH may serve as WM training.

Our finding of CCH’s benefit to inhibition control is novel, but consistent with the indirect evidence that CCH training reduced ADHD symptoms [17]. Indeed, CCH as well as Chinese painting is commonly believed to increase patience. Contrary to our hypothesis, CCH was not associated with the shifting ability. Perhaps shifting is less crucial to CCH. Nevertheless, brain regions related to shifting (left IFJ in particular) did show stronger RSFCs in the CCH group than the control group. One possible explanation for the apparent inconsistent result between behavioral and imaging data is that brain activities are more sensitive than the behavioral measures as indices of training effects [28, 65].

Indeed, RSFC between all but one seed regions showed stronger RSFC for the CCH group than the control group. First, the bilateral dlPFC seed showed stronger RSFC with the parietal lobe (PstCG, PCG and precuneus), which were consisted with the fronto-parietal network involved in WM [28, 66]. WM training has also been found to strengthen the RSFC between the frontal gyrus and other brain areas included in the fronto-parietal network [67]. Second, the CCH group showed stronger RSFC between left SFC seed and AG, ACC, and MOFC, brain areas that have been found to play a critical role in EF [68]. Interestingly, the right SFC seed did not show any significant results. Indeed, previous research has shown that compared to the right SFC, the left SFC is more critical for WM [69] [70, 71]. Third, the frontal lobe also showed stronger RSFC with the temporal lobe (STG, MTG, ITG and HG), which has been found to play important roles in memory function [72, 73]. Fourth, consistent with the literature on the importance of the IFG in response inhibition [74–76], we found that CCH was associated with stronger RSFCs between the IFG and parietal lobe (SPG, PstCG, precuneus). Moreover, there was a positive correlation between STG-related RSFC and the behavior index of inhibition (i.e. VE_250).

Finally, the ACC has been found to play an important role in all three aspects of EF [77–80]. We found stronger RSFC for the CCH group between the ACC seed and brain areas involved in VPN and SPN, which might explain previous findings of CCH’s role in improving visual attention and perception [17]. RSFCs between ACC and several other areas (IFG, cuneus and caudate) were also stronger for the CCH group, which was consistent with the important roles of the PFC and basal ganglia in EF [75, 81–83].

Several limitations of the current study need to be mentioned. First, it was a correlational study. Although resource-intensive, a prospective longitudinal study tracking the training of CCH across many years would provide more direct support for its benefits to EFs. Second, the sample size was very small and the effect sizes were modest. Third, the present study used only three sub-tasks to assess EFs. More tasks can be used in future research.

## 6. Conclusion

The current study demonstrated that long-term CCH training was associated with better executive functions and stronger RSFC of the frontal and parietal cortex and basal ganglia.

## Supporting Information

S1 Table Behavioral measures of the cue-target paradigm task (VE_50 and VE_250), Corsi block test (forward score and backward score), and Stroop color-word test (IS time and IE difference score). The **‘**VE_50**’**means validity effect of SOA_50; the ‘VE_250’ means validity effect of SOA_250; the ‘IS time’ means the interference score for time; and the ‘IE’ means inverse efficiency score. ‘IE difference score’ was calculated by subtracting the color-naming trials’ IE from the incongruent trials’ IE.
(XLSX)

S2 Table Averaged RSFC of brain areas showing significant correlation with beahavioral measures. The ‘L’ means left hemisphere; The ‘R’ means right hemisphere; the ‘FFG’ means fusiform gyrus; the ‘AG’ means angular gyrus; the ‘STG’ superior temporal gyrus; the ‘ACC’ means anterior cingulate cortex; and the ‘SOG’ means superior occipital gyrus.
(XLSX)
